# Effectiveness of Hypoglossal Nerve Stimulation Therapy in Positional Obstructive Sleep Apnea: A Retrospective Observational Study

**DOI:** 10.3390/jcm14165873

**Published:** 2025-08-20

**Authors:** Lidice Hernandez, Daniela Trelles-Garcia, Michael Medina, Kaylee Sarna, Laurence Smolley, Anas Hadeh

**Affiliations:** Cleveland Clinic Florida, Weston, FL 33331, USA; trelled@ccf.org (D.T.-G.); medinam2@ccf.org (M.M.); sarnak@ccf.org (K.S.); smollel@ccf.org (L.S.);

**Keywords:** hypoglossal nerve stimulation, obstructive sleep apnea, positional sleep apnea, apnea–hypopnea index, sleep architecture, excessive day time hypersomnolence, polysomnography

## Abstract

**Background**: Obstructive sleep apnea (OSA) is a prevalent disorder characterized by airway collapse during sleep. Continuous positive airway pressure (CPAP) is first-line treatment but adherence can decay over time due to intolerance. Hypoglossal nerve stimulation (HNS) has emerged as an alternative, especially for CPAP-intolerant patients. OSA can be classified into position-dependent (PD-OSA) and non-position-dependent (NPD-OSA) subtypes based on apnea–hypopnea index (AHI) variation by sleep posture. **Study Objectives**: This study aims to evaluate polysomnographic changes following HNS therapy and compare treatment outcomes in PD-OSA and NPD-OSA patients. **Methods**: A retrospective observational study of 30 patients treated with HNS at a single center between January 2022 and March 2025 was conducted. The primary endpoint was change in overall apnea–hypopnea index (AHI) from baseline to first post-implant in-laboratory polysomnography (PSG). Secondary endpoints included changes in phenotype-specific (supine and non-supine) AHI, Epworth Sleepiness Scale (ESS), and sleep architecture parameters. Subgroup comparisons were performed between PD-OSA and NPD-OSA phenotypes. **Results**: Thirty patients (median age 69.5 years; 73% male; median BMI 28.9 kg/m^2^) were included; 27 had sufficient positional data for phenotype classification (66.7% PD-OSA, 33.3% NPD-OSA). Median AHI decreased from 23.5 to 4.8 events/h (*p* < 0.0001), with reductions in both REM and supine AHI. PD-OSA patients demonstrated the greatest improvement in supine AHI, whereas NPD-OSA patients uniquely improved in non-supine AHI. ESS decreased by a median of 1.5 points overall (*p* = 0.0015) and met the minimal clinically important difference in NPD-OSA. Sleep architecture showed minimal change, except for a reduction in supine sleep percentage (*p* = 0.0114). **Conclusions**: HNS therapy improved AHI and subjective sleepiness across OSA phenotypes, with distinct positional responses. These findings support the clinical utility of HNS in both PD-OSA and NPD-OSA and suggest phenotype-specific treatment effects warrant further investigation.

## 1. Introduction

Obstructive sleep apnea (OSA) is the most common sleep-related breathing disorder, affecting approximately 15–30% of males and 10–15% of females in North America [1]. Characterized by repetitive airway collapse during sleep, untreated OSA leads to increased cardiovascular and neurological morbidity. Additionally, quality of life is affected as these obstructions commonly manifest as excessive daytime sleepiness, loud snoring and difficulty concentrating [2].

A subset of patients exhibits a positional phenotype, known as positional OSA (PD-OSA in this study), where respiratory events are more frequent or severe when the individual sleeps in the supine position [3,4]. One study estimates that up to 56% of OSA patients demonstrate this positional dependence [5]. The distinction between positional and non-positional OSA (NPD-OSA in this study) is clinically relevant, as it may guide therapeutic decision-making. For example, positional therapy—encouraging patients to avoid sleeping supine—can be effective in selected cases of PD-OSA, while its utility in NPD-OSA is limited [3,6,7].

Regardless of phenotype, continuous positive airway pressure (CPAP) therapy is the gold standard treatment, but poor tolerance and adherence limit its effectiveness in many patients. Patients often find CPAP therapy intolerable due to discomfort from the mask, air leaks, dryness, and claustrophobia, leading to poor adherence [2,8]. Consequently, patients seeking alternative treatments may opt for hypoglossal nerve stimulation, which offers a novel treatment option [9,10]. Hypoglossal nerve stimulation (HNS) therapy offers an alternative by electrically activating the hypoglossal nerve to protrude and stiffen the tongue, reducing upper airway collapse during sleep [9,11]. The STAR trial demonstrated significant reductions in apnea–hypopnea index (AHI) and improvement in subjective sleep measures at 12 months of HNS therapy [9]. Numerous other studies have demonstrated the efficacy of HNS in reducing the apnea–hypopnea index (AHI) and improving quality of life [10,12,13]. Nonetheless, it remains unclear whether the response to HNS differs between PD-OSA and NPD-OSA patients.

The underlying pathophysiology of PD-OSA is thought to reflect a greater dependency on gravitational airway collapse during supine sleep, whereas NPD-OSA may involve more anatomically or neuromuscular fixed upper airway obstruction that persists across all sleep positions [3,14,15]. These differences suggest that patients with PD-OSA may have more positional airway collapsibility that is directly addressed by HNS through targeted tongue protrusion and upper airway stabilization. Conversely, NPD-OSA patients may exhibit more generalized or multilevel airway compromise, potentially leading to broader positional improvements post-HNS [11,16,17]. These mechanistic differences form the rationale for exploring phenotype-specific responses to HNS therapy.

Upon review of the current literature, only one study that included 46 participants suggested that patients with positional OSA may experience greater improvements in AHI following HNS therapy compared to those with non-positional OSA [18]. However, this study was presented in abstract form only, with limited methodological detail and statistical reporting, which constrains the ability to draw firm conclusions.

Given these limitations in the literature, this study aims to compare the effects of HNS therapy on polysomnographic parameters within and between patients with positional and non-positional OSA. Polysomnography (PSG) remains the gold standard for both OSA diagnosis and positional phenotype classification, and it enables precise quantification of supine- and non-supine-specific treatment effects in a real-world setting.

Specifically, we assess changes in supine and non-supine AHI before and after treatment to determine whether positional phenotype influences treatment response. We hypothesized that HNS would result in significant improvements in respiratory indices for both PD-OSA and NPD-OSA, but that phenotype-specific differences in supine and non-supine AHI responses would be observed. The primary aim was to assess changes in overall AHI from baseline to post-treatment PSG. Secondary aims were to (1) evaluate phenotype-specific changes in supine and non-supine AHI, (2) assess subjective sleepiness via the Epworth Sleepiness Scale, and (3) examine changes in sleep architecture.

## 2. Materials and Methods

This retrospective observational study included 30 patients diagnosed with obstructive sleep apnea from the Cleveland Clinic Healthcare System in Weston, FL, who underwent implantation of a hypoglossal nerve stimulator between 1 January 2022 and 1 March 2025.

### 2.1. Selection Criteria and Diagnostic Categories

Patient selection followed standard clinical criteria for HNS therapy at our institution. Inclusion criteria were: age ≥ 18 years, polysomnographic diagnosis of moderate to severe OSA with a central apnea index (CAI) < 25%, pre-implantation apnea–hypopnea index (AHI) between 15 and 65 events/h, body mass index (BMI) < 32 kg/m^2^, intolerance or refusal of CPAP therapy, and anterior–posterior predominant retropalatal collapse observed during drug-induced sleep endoscopy. The BMI < 32 kg/m^2^ threshold was selected in accordance with inclusion criteria from the STAR trial and other pivotal HNS studies, which demonstrated optimal treatment response and lower complication rates in patients within this range [9]. Exclusion criteria included significant upper airway anatomical abnormalities, history of neuromuscular disease, hypoglossal nerve palsy, and severe cardiopulmonary disease. The dataset included 45 patients originally. 30 patients were included in the final analysis after noted exclusions were removed from the dataset.

Patients included in the study all underwent polysomnography prior to implantation of device. All patients underwent repeat polysomnography at least one-month post-implantation. All PSGs were performed in an AASM-accredited sleep laboratory and were manually scored by registered polysomnographic technologists according to the 2012 AASM scoring criteria. Hypopneas were defined as ≥30% reduction in airflow for ≥10 s associated with ≥3% oxygen desaturation or an arousal. Data extracted from chart review included age, sex, pre- and post-implantation BMI, prior OSA treatment history, and therapy-related complications. Polysomnographic parameters analyzed included sleep architecture; Total Sleep Time (TST), sleep efficiency, awakenings, arousal index, sleep latency, REM latency, body position percentages, stage shifts) and respiratory variables (Total AHI, Supine AHI, Off-Supine AHI, REM and NREM AHI, oxygen saturation nadir and median oxygen saturation, time spent with oxygen saturation less than 90%. Additionally, ESS scores were documented before implantation as well as during follow up outpatient visits after complete titration of hypoglossal nerve stimulator.

Documentation regarding whether patients received formal positional therapy or sleep posture counseling was unavailable; as such, this variable could not be assessed.

### 2.2. Positional OSA Classification

Patients were classified as PD-OSA if the pre-treatment supine AHI was at least twice the off-supine AHI [10]. Those not meeting this criterion were classified as NPD-OSA. Patients without position data on either pre or post treatment polysomnography were not classified.

### 2.3. Statistical Analysis

Descriptives of participants were looked at in the general dataset. Categorical variables were measured with counts and frequencies. Data distribution was assessed using the Shapiro–Wilk test; non-normal variables were analyzed using non-parametric methods. Chi square and Fisher exact tests for smaller sample sizes were performed to evaluate the association between pre-post variables. Medians, interquartile ranges, and first and third quartile values were recorded for continuous variables and difference variables of post–pre variable calculated. Wilcoxon signed rank tests were performed to determine statistically significant differences between OSA position-dependent groups. Analyses for the overall cohort were conducted using the full sample of 30 patients. Subgroup analyses by phenotype (PD-OSA vs. NPD-OSA) were limited to the 27 patients with available positional data. Missing data were handled by listwise deletion for each specific analysis; no imputation was performed. *p* values of <0.05 were determined to be statistically significant in this analysis. SAS 9.4 and R 4.4.1 were used to analyze the data. Wilcoxon signed rank tests were performed to determine statistically significant differences between OSA position dependent groups.

Given the exploratory and hypothesis-generating nature of the study, no formal adjustment for multiple comparisons was applied. We acknowledge that this approach may increase the risk of type I error, and findings should be interpreted accordingly.

This study was deemed Exempt Human Subjects Research by the Institutional Review Board IRB# FLA 25-214. This study was not registered as a clinical trial, as it was a retrospective observational analysis and did not meet trial registration criteria.

## 3. Results

### 3.1. Participant Characteristics

After exclusions, 30 patients were included in the overall analysis (Table 1). Of these, 27 had sufficient positional data for phenotype classification into PD-OSA or NPD-OSA. All subgroup analyses are based on this subset of 27 patients. There were more position dependent OSA patients than not (66.7% vs. 33.3%). Median age was 69.5 years (IQR 17.0); 73.3% were male. Median BMI was 28.9 (IQR 6.5). 90% had prior CPAP experience. Median time from implantation to follow-up PSG was 270.5 days (IQR 189).

### 3.2. Overall Outcomes in Sleep Architecture

Sleep architecture remained largely stable following hypoglossal nerve stimulation therapy. There were no statistically significant changes in total sleep time (median change: +7.0 min, *p* = 0.6501) or sleep efficiency (median change: +1.6%, *p* = 0.9580). Similarly, no significant differences were observed in sleep latency, REM latency, number of awakenings, or time awake after sleep onset. The proportion of time spent in each sleep stage (N1, N2, N3, and REM) remained consistent across pre- and post-treatment. REM sleep percentage remained unchanged (median change: −0.7%, *p* = 0.2588).

Although not reaching statistical significance, trends were noted toward improvement in arousal-related metrics. The number of arousals decreased (median change: −34 events, *p* = 0.0504), and the arousal index trended lower (median change: −5.0 events/h, *p* = 0.0833). While these borderline *p*-values do not meet the conventional threshold for significance, such reductions may still be clinically meaningful in terms of sleep continuity and patient-reported quality of rest, and warrant further investigation in larger cohorts.

Importantly, the only variable to demonstrate a statistically significant change was the percentage of total sleep time spent in the supine position, which decreased from a median of 56.7% to 36.7% post-treatment (*p* = 0.0114). Although documentation on positional counseling was unavailable, this reduction may reflect spontaneous behavioral changes or improved sleep comfort following HNS therapy.

Subjective sleep quality, as measured by ESS, improved significantly, with scores decreasing from a median of 8.0 to 6.0 (*p* = 0.0015), indicating reduced daytime sleepiness post-treatment with a difference calculation of 1.5 (Table 2).

### 3.3. Overall Outcomes in Respiratory and Subjective Variables

Hypoglossal nerve stimulation therapy was associated with significant improvements in multiple respiratory parameters among all participants. The total number of apneas decreased markedly from a median of 30.0 to 2.0 events per night (*p* = 0.0002), with a similar reduction observed in obstructive apneas (median: 27.0 to 1.0, *p* < 0.0001). There were no significant changes in mixed or central apneas, consistent with the exclusion of patients with a central apnea index ≥ 25 from the study cohort.

Hypopneas showed a non-significant downward trend, decreasing from a median of 79.0 to 28.0 events (*p* = 0.0967). Importantly, the total apnea–hypopnea index (AHI) improved significantly, with a median reduction from 23.5 to 4.8 events/h (*p* < 0.0001). This reduction was observed across both REM and NREM sleep stages. Total REM AHI dropped significantly from 33.5 to 7.1 events/h (*p* = 0.0004), while data for total NREM AHI was too limited to draw conclusions (available in only two post-treatment studies, *p* = 1.000). The very small number of patients with complete NREM data post-treatment limits statistical power for this sub-analysis and may introduce bias; these findings should therefore be interpreted with caution.

Significant improvements were also seen in positional respiratory indices. Supine total AHI decreased from 43.9 to 12.3 events/h (*p* < 0.0001), and supine REM AHI decreased from 46.4 to 10.9 events/h (*p* = 0.0076). In contrast, off-supine total and REM AHIs showed non-significant changes.

Oxygenation metrics also trended toward improvement. Mean oxygen saturation increased slightly (median: 94.0% to 95.0%, *p* = 0.0719), and minimum oxygen saturation improved from 81.0% to 84.0% (*p* = 0.1435). Although these differences were not statistically significant, even modest improvements in oxygen nadir can have potential clinical relevance, particularly in patients with cardiovascular comorbidities. Time spent with oxygen saturation < 90% decreased from 1.9% to 0.5% of total sleep time, though this did not reach statistical significance (*p* = 0.1873) (Table 3).

### 3.4. Outcomes in Position Dependent-OSA

Among the 18 PD-OSA patients within the 27-patient positional dataset, HNS therapy led to significant improvements in key respiratory parameters. The median total AHI decreased from 22.6 (IQR: 16.6–30.5) to 4.5 (IQR: 3.0–8.3) events/h, with a statistically significant median reduction of –17.6 (IQR: −22.3 to −11.2; *p* = 0.0011). Improvements were also observed in REM AHI (from 28.3 to 11.6, *p* = 0.0244) and supine AHI (from 47.5 to 12.6, *p* = 0.0103).

While changes in off-supine AHI and NREM AHI were not statistically significant, the reduction in obstructive apneas (from 36.0 to 3.0, *p* = 0.0040) and total apneas (from 37.0 to 3.0, *p* = 0.0110). Minimum oxygen saturation improved modestly, and subjective sleepiness (ESS score) decreased from a median of 7.5 to 5.5 (−1.5; *p* = 0.0625), approaching statistical significance.

Sleep architecture showed nonsignificant trends toward fewer arousals (−47.0, *p* = 0.0460), lower arousal index (−9.1, *p* = 0.0638), although total sleep time and efficiency remained stable. Time spent supine during sleep did not differ significantly after treatment (Table 4).

### 3.5. Outcomes in NPD-OSA Group

In the 9 NPD-OSA patients within the 27-patient positional dataset, treatment with HNS also yielded substantial improvements in respiratory metrics. The median total AHI significantly declined from 32.0 (IQR: 16.8–40.8) to 4.1 (IQR: 0.6–14.2), with a median reduction of –18.2 (IQR: −40.2 to −14.9; *p* = 0.0078). Supine total AHI improved from 34.0 to 10.8 (−22.5; *p* = 0.0078), and REM AHI trended downward from 45.6 to 0.8 events/h (−40.4; *p* = 0.0625).

The number of obstructive apneas significantly decreased (from 15.0 to 0.0; *p* = 0.0156), as did total apneas (−15.5; *p* = 0.0156). The number of hypopneas also decreased notably (−77.0), though not reaching statistical significance (*p* = 0.1641).

Subjective sleepiness (ESS) improved significantly in NPD-OSA patients, with scores decreasing from 10.0 to 6.0 (−3.0; *p* = 0.0156). However, changes in sleep architecture were less favorable compared to the PD-OSA group: total awakenings increased (*p* = 0.0156), and total time awake after sleep onset trended upward, though not significantly. The percentage of time spent supine during sleep decreased significantly (from 76.0% to 31.3%; *p* = 0.0313) (Table 5).

### 3.6. Between-Group Comparison of Key Respiratory Variables

Patients with NPD-OSA had significantly higher off-supine total AHI compared to PD-OSA patients (median 30.9 [IQR: 13.5–34.0] vs. 7.5 [IQR: 4.5–10.0]; *p* = 0.0021). Similarly, off-supine NREM AHI was significantly higher in the NPD-OSA group than in the PD-OSA group (median 21.6 [IQR: 10.0–34.0] vs. 6.1 [IQR: 3.9–10.1]; *p* = 0.0149). These differences are consistent with their respective phenotypic definitions and support the distinction between the two groups.

Both subgroups experienced significant reductions in AHI following hypoglossal nerve stimulation (HNS) therapy. Supine total AHI improved in both NPD-OSA and PD-OSA patients (median change: −22.5 vs. −24.2; *p* = 0.9743), and there were no statistically significant differences in the reduction of supine REM AHI or supine NREM AHI between the groups.

However, the reduction in off-supine total AHI was significantly greater in the NPD-OSA group (median change: −11.1 [IQR: −31.2 to −9.1]) compared to the PD-OSA group (median change: 0.2 [IQR: −4.3 to 7.5]; *p* = 0.0216), suggesting a broader therapeutic benefit for NPD-OSA patients across all sleeping positions. Changes in off-supine REM and NREM AHI did not reach statistical significance, likely due to small sample sizes.

Reductions in overall total AHI, total REM AHI, and total NREM AHI were observed in both groups, with no significant between-group differences (e.g., total AHI change: −18.2 in NPD-OSA vs. −17.6 in PD-OSA; *p* = 0.3512). Notably, total REM AHI decreased more in the NPD-OSA group (−40.4) than in the PD-OSA group (−18.5), though this difference did not reach statistical significance (*p* = 0.0699) (Table 6).

## 4. Discussion

Obstructive sleep apnea (OSA) is a prevalent condition affecting an estimated 26% of adults in the U.S., with increasing incidence due to rising rates of obesity and aging populations [1]. While continuous positive airway pressure (CPAP) therapy remains the first-line treatment, its limited adherence across age groups has prompted investigation into alternative therapies, including hypoglossal nerve stimulation (HNS) [2,8]. This retrospective study evaluates real-world outcomes of HNS using pre- and post-treatment polysomnography (PSG), specifically comparing clinical responses in patients with positional (PD-OSA) and non-positional OSA (NPD-OSA). Our findings support the broad efficacy of HNS and highlight phenotypic variability in treatment response.

The study cohort consisted predominantly of older adults with a median age of 69.5 years and a median BMI within the overweight range. The majority of patients were male and had prior experience with CPAP therapy, reflecting a population representative of those commonly evaluated for alternative OSA treatments such as hypoglossal nerve stimulation. Nearly nine months elapsed between implantation and post-treatment polysomnography, allowing sufficient time for therapeutic stabilization. Among the 27 patients with available positional data, a greater proportion were classified as having position-dependent OSA (66.7%) compared to non-position-dependent OSA (33.3%). This distribution aligns with prior literature suggesting that positional OSA comprises a substantial subset of the OSA population [3,7] and underscores the relevance of investigating treatment responses within these phenotypes.

Consistent with previous randomized trials and meta-analyses [9,12,15], we observed significant overall reductions in total AHI following HNS therapy (median reduction: −17.6 events/h), with marked improvements in obstructive and total apneas. These improvements extended across REM and NREM stages, affirming the multi-phase efficacy of the therapy. Supine-specific improvements were notable, including reductions in supine AHI (−25.5), supine REM AHI (−29.0), and supine NREM AHI. These reductions align with prior work suggesting that supine-dominant obstruction can be effectively mitigated by upper airway stimulation [13,16]. In contrast, non-supine AHI did not significantly change in the full cohort, likely reflecting both the lower baseline severity in this posture and the targeted nature of HNS therapy, which may primarily affect airway collapsibility in the supine position.

Sleep architecture was largely preserved post-treatment. Measures such as total sleep time, sleep efficiency, and REM sleep percentage showed no statistically significant change. However, favorable trends were observed in arousal-related metrics, with a near-significant decrease in arousals (–34 events; *p* = 0.0504) and arousal index (−5.0 events/h; *p* = 0.0833). Similarly, minimum oxygen saturation and mean oxygen saturation demonstrated non-significant improvements. Given the relatively small sample size and variability in baseline oxygenation, these findings may reflect limited statistical power rather than an absence of a physiological effect, and could become significant in larger cohorts. These findings suggest that HNS may not disrupt sleep continuity and may even modestly improve sleep stability—an important clinical consideration given the bidirectional relationship between arousals and perceived sleep quality.

In our study, the overall median Epworth Sleepiness Scale (ESS) score improved from 8.0 to 6.0, with a median difference of −1.5 points (*p* = 0.0015). Among patients with non-positional obstructive sleep apnea (NPD-OSA), ESS scores improved significantly from 10.0 to 6.0, with a median difference of −3.0 (*p* = 0.0156). In the positional OSA (PD-OSA) subgroup, ESS scores decreased from 7.5 to 5.5, with a median difference of −2.0 (*p* = 0.0625). These reductions are consistent with or exceed the minimum important difference (MID) of 2.0 points estimated by a previous study [17], which represents a threshold for meaningful clinical improvement in daytime sleepiness. The finding reinforces the clinical relevance of hypoglossal nerve stimulation (HNS) therapy in improving not just objective respiratory metrics, but also patient-perceived outcomes across both positional and non-positional OSA populations. These findings suggest that HNS may be particularly effective in alleviating excessive daytime sleepiness in patients with more generalized, non-positional forms of OSA. Importantly, the percentage of time spent sleeping supine decreased significantly post-treatment (−20.0%; *p* = 0.0114), possibly reflecting behavioral adaptation or indirect effects of improved airway patency during supine sleep.

When stratified by phenotype, both PD-OSA and NPD-OSA groups demonstrated significant improvements in total AHI, supine AHI, and apneas. Notably, NPD-OSA patients exhibited significantly greater improvement in off-supine AHI (−11.1 vs. 0.2; *p* = 0.0216), suggesting broader positional responsiveness to HNS therapy in this group. This broader positional improvement in NPD-OSA patients may reflect a more generalized reduction in airway collapsibility across sleep positions, potentially due to multilevel obstruction or neuromuscular factors that are responsive to HNS regardless of posture. Conversely, PD-OSA patients demonstrated their greatest gains in supine-specific metrics, aligning with the gravitational and anatomic influences that define this phenotype. While REM AHI decreased more in NPD-OSA patients (−40.4 vs. −18.5), this difference did not reach statistical significance. These subgroup analyses reinforce the phenotypic distinctions originally described by Cartwright (1984) and later elaborated in studies of positional classification and OSA severity [3,4,7].

Compared to the only previous similar study [14], our study adds clinical value by including only in-lab PSG studies, providing more detailed and controlled measurements of sleep architecture, positional effects, and staging data. While the former study found that HNS improved outcomes in PD-OSA, their findings were reported in abstract form and based primarily on home sleep apnea testing [14]. By contrast, our analysis allows for more granular, stage-specific, and positional interpretation and provides longer-term follow-up (median: 270.5 days).

Although our results are encouraging, several limitations warrant consideration. The study was retrospective observational design without randomization or a control group, which limits the level of evidence. Additionally, the study was limited by a small sample size (n = 30), particularly in the subgroup analyses, where some post-treatment metrics (e.g., NREM AHI) were available in only a few patients. This limited availability likely reduced power to detect meaningful differences, especially in outcomes with higher variability such as oxygenation metrics. Only 27 of the 30 patients had sufficient positional data to allow for classification into PD-OSA or NPD-OSA groups. This further reduced the subgroup sample size and may have limited statistical power for between-group comparisons. The lack of a control group also limits our ability to attribute changes solely to HNS therapy. In addition, 15 patients were excluded due to incomplete follow-up or inadequate positional data, which may introduce selection bias if their outcomes systematically differed from those included. We also lacked information on potential confounders, including interim weight changes, concurrent surgical procedures, or adherence to HNS therapy, all of which could influence treatment response. Future prospective studies with larger, more diverse cohorts and comprehensive follow-up data are needed to address these gaps and validate our findings.

Furthermore, we restricted inclusion to patients with a BMI < 32 kg/m^2^, which reflects established HNS candidacy guidelines and eligibility criteria used in foundational trials such as STAR [9]. While this threshold was selected to optimize treatment efficacy and minimize procedural risk, it may limit the generalizability of our findings to individuals with more severe obesity. Notably, emerging evidence suggests that HNS may be beneficial in select patients with BMI ≥ 32 kg/m^2^, warranting further investigation in this subgroup [19]. The cohort was predominantly older, male, and non-obese, reflecting current HNS candidacy trends. These characteristics may limit the generalizability of our findings to younger patients, females, or individuals with higher BMI outside standard inclusion criteria.

Additionally, no correction for multiple comparisons was performed. While this approach preserved sensitivity in the context of a small exploratory sample, it may have increased the likelihood of type I error across the numerous outcomes assessed. Finally, while ESS scores improved, other patient-reported outcomes such as snoring, fatigue, or cardiovascular endpoints were not assessed.

From a clinical care perspective, these findings support the inclusion of both PD-OSA and NPD-OSA patients in the candidate pool for HNS evaluation and suggest that phenotypic classification should inform—but not restrict—referral patterns. Incorporating routine positional analysis into pre-implant PSG could help tailor patient counseling and manage expectations, while structured follow-up with in-lab PSG may help optimize device settings and track longitudinal efficacy. Broader adoption of these strategies could facilitate guideline refinement and integration of HNS into multidisciplinary sleep medicine workflows.

Despite these limitations, the real-world nature of our study enhances its clinical relevance. All patients were managed in a standard care setting and underwent comprehensive pre- and post-treatment PSG, providing insight into how HNS performs outside of controlled trials. These findings support the inclusion of both PD-OSA and NPD-OSA patients in the candidate pool for HNS and suggest that positional phenotype should not preclude referral for upper airway stimulation evaluation.

The results provide a foundation for future prospective, multi-center studies with larger and more diverse populations, standardized positional phenotyping, and comprehensive data on device adherence, stimulation parameters, and patient-reported outcomes. Further work should also examine long-term cardiovascular, metabolic, and quality-of-life endpoints, as well as cost-effectiveness, to better inform clinical guidelines and optimize care pathways for both PD-OSA and NPD-OSA patients.

## Figures and Tables

**Table 1:** Patient descriptives for entire population (n = 30).

Variable	Median (n = 30)
Age, years	69.5 [17.0, 57.0–74.0]
Sex, N (%)	
Female	8 (26.7)
Male	22 (73.3)
BMI	28.9 [6.5, 21.6–35.3]

**Table 2:** Overall sleep architecture variables for pre- and post-treatment (n = 30).

Variable Median (IQR, Q1–Q3)	Pre-Treatment	Post-Treatment	Difference Calculation (Post–Pre Treatment)	*p* Value
Total sleep time	341.0 [84.0, 282.0–366.0], n = 30	320.0 [87.0, 289.0–376.0], n = 29	7.0 [62.0, −20.0–42.0], n = 29	0.6501
Sleep efficiency	82.7 [18.6, 67.4–86.0], n = 30	79.3 [16.0, 70.2–86.2], n = 29	1.6 [8.9, −5.4–3.5], n = 29	0.9580
Total awakenings	24.5 [22.0, 16.0–38.0], n = 26	24.0 [25.0, 17.0–42.0], n = 29	3.5 [23.0, −13.0–10.0], n = 26	0.4797
Total time awake after sleep onset	51.8 [76.8, 19.0–95.8], n = 28	65.5 [68.0, 36.0–104.0], n = 29	12.5 [56.0, −12.5–43.5], n = 27	0.1475
Sleep latency	10.8 [25.0, 5.5–30.5], n = 30	14.8 [12.8, 7.0–19.8], n = 28	1.0 [21.8, −13.3–8.5], n = 28	0.6314
REM latency	118.0 [110.0, 92.0–202.0], n = 30	102.5 [132.0, 63.5–195.5], n = 28	−19.0 [139.0, −88.5–50.5], n = 28	0.4222
Total amount in supine	56.7 [42.8, 38.2–81.0], n = 24	36.7 [47.2, 19.1–66.3], n = 29	−10.0 [39.2, −34.2–5.0], n = 24	0.0114
Stage N1	12.4 [11.9, 9.2–21.1], n = 28	14.0 [9.0, 8.9–17.9], n = 29	1.6 [14.2, −6.2–8.0], n = 28	0.6733
Stage N2	63.7 [14.7, 53.9–68.6], n = 29	64.9 [11.4, 57.8–69.2], n = 29	0.4 [19.5, −6.1–13.3], n = 28	0.4093
Stage N3	3.0 [5.1, 0.0–5.1], n = 29	0.4 [5.3, 0.0–5.3], n = 29	−0.3 [4.5, −4.0–0.5], n = 28	0.2155
% in REM	16.5 [10.0, 12.4–22.4], n = 30	15.2 [7.6, 12.2–19.8], n = 29	−0.7 [12.4, −8.2–4.2], n = 29	0.2588
Number of arousals	158.0 [88.0, 99.0–187.0], n = 26	139.0 [113.0, 63.0–176.0], n = 29	−34.0 [97.0, −77.0–20.0], n = 26	0.0504
Arousal index	30.1 [15.3, 19.8–35.1], n = 29	24.8 [24.5, 11.8–36.3], n = 29	−5.0 [19.1, −15.7–3.5], n = 28	0.0833
Stage shifts	128.0 [56.0, 85.0–141.0], n = 23	86.0 [53.0, 70.0–123.0], n = 29	−30.0 [65.0, −48.0–17.0], n = 23	0.1155
ESS	8.0 [7.0, 5.0–12.0], n = 26	6.0 [8.0, 3.0–11.0], n = 30	−1.5 [4.0, −4.0–0.0], n = 26	0.0015

**Table 3:** Overall respiratory and subjective variables for pre- and post-treatment (n = 30).

Variable Median (IQR, Q1–Q3), n = 29	Pre-Treatment (n = 30)	Post-Treatment (n = 30)	Difference Calculation (Post–Pre Treatment) (n = 30)	*p* Value
Number of apneas	30.0 [59.0, 14.0–73.0], n = 25	2.0 [8.0, 0.0–8.0], n = 29	−22.0 [54.0, −59.0–−5.0], n = 25	0.0002
Number of obstructive apneas	27.0 [60.0, 11.0–71.0], n = 27	1.0 [5.0, 0.0–5.0], n = 29	−15.0 [39.0, −42.0–−3.0], n = 27	<0.0001
Number of hypoapneas	79.0 [66.0, 28.0–94.0], n = 27	28.0 [76.0, 14.5–90.5], n = 28	−44.0 [91.0, −70.0–21.0], n = 27	0.0967
Total AHI	23.5 [17.6, 16.6–34.2], n = 30	4.8 [6.9, 1.7–8.6], n = 28	−17.6 [15.4, −26.8–−11.4], n = 28	<0.0001
Total REM AHI	33.5 [28.3, 23.4–51.7], n = 23	7.1 [12.0, 0.0–12.0], n = 20	−30.0 [24.7, −40.4–−15.7], n = 17	0.0004
Total NREM AHI	21.0 [19.7, 13.1–32.8], n = 22	27.1 [54.2, 0.0–54.2], n = 2	4.4 [34.0, −12.6–21.4], n = 2	1.000
Supine total AHI	43.9 [29.0, 25.6–54.6], n = 29	12.3 [13.7, 6.1–19.8], n = 25	−25.5 [31.7, −42.7–−11.0], n = 24	<0.0001
Supine REM AHI	46.4(30.6, 29.4–60.0), n = 19	10.9 [31.6, 0.0–31.6], n = 21	−29.0 [46.4, −51.7–−5.3], n = 16	0.0076
Supine NREM AHI	44.7 [33.3, 18.3–51.6], n = 22	0.0 [53.4, 0.0–53.4], n = 3	−1.1 [24.4, −13.3–11.1, n = 2	1.000
Off supine total AHI	8.8 [8.8, 6.0–14.8], n = 25	5.1 [11.4, 0.7–12.0], n = 16	−4.3 [17.5, −10.0–7.5], n = 15	0.4887
Off supine REM AHI	20.2 [24.0, 7.1–31.1], n = 18	0.0 [0.0, 0.0–0.0], n = 12	−7.1 [38.7, −32.9–5.8], n = 7	0.3125
Off supine NREM AHI	7.9 [7.0, 4.7–11.7], n = 20	27.8 [55.5, 0.0–55.5], n = 2	19.2 [58.4, −10.0–48.4], n = 2	1.000
Mean oxygen saturation	94.0 [3.0, 93.0–96.0], n = 27	95.0 [2.0, 94.0–96.0], n = 29	0.0 [1.0, 0.0–1.0], n = 26	0.0719
Minimum oxygen saturation	81.0 [11.0, 75.0–86.0], n = 29	84.0 [10.0, 79.0–89.0], n = 29	3.0 [11.5, −2.5–9.0], n = 28	0.1435
Sleep time with oxygen saturation below 90%	1.9 [5.7, 0.3–6.0], n = 25	0.5 [3.3, 0.0–3.3], n = 29	−0.3 [2.6, −2.4–0.2], n = 25	0.1873

**Table 4:** Sleep study variables for pre- and post-treatment in PD-OSA (n = 18 of the 27 patients with positional data).

Variable	Pre-Treatment (n = 18)	Post-Treatment (n = 18)	Difference Calculation (Post–Pre Treatment) (n = 18)	*p* Value
Total sleep time, median(IQR, Q1–Q3), n = 29	321.0 [64.0, 282.0–346.0], n = 17	319.0 [100.0, 289.0–389.0], n = 17	−2.0 [61.0, −20.0–41.0], n = 17	0.9540
Sleep efficiency, median(IQR, Q1–Q3)	76.7 [15.6, 67.4–83.0], n = 17	78.8 [16.0, 70.2–86.2], n = 17	2.0 [9.3, −3.4–5.9], n = 17	0.6777
Total awakenings, median(IQR, Q1–Q3)	27.0 [26.0, 16.0–42.0], n = 17	22.0 [32.0, 12.0–44.0], n = 17	−4.0 [29.0, −19.0–10.0], n = 17	0.5398
Total time awake after sleep onset, median(IQR, Q1–Q3)	64.5 [58.0, 39.0–97.0], n = 17	71.5 [65.0, 39.0–104.0], n = 17	−8.0 [42.0, −16.5–25.5], n = 17	1.000
Sleep latency, median(IQR, Q1–Q3)	10.8 [30.5, 5.5–36.0], n = 17	15.0 [12.5, 7.0–19.5], n = 17	3.5 [20.5, −12.0–8.5], n = 17	0.8634
REM latency, median(IQR, Q1–Q3)	118.0 [97.0, 105.0–202.0], n = 17	109.0 [111.0, 71.0–182.0], n = 17	−12.0 [121.0, −82.0–39.0], n = 17	0.4038
Total amount in supine, median(IQR, Q1–Q3)	40.3 [30.1, 33.4–63.4], n = 16	37.0 [42.2, 19.5–61.7], n = 17	−4.0 [37.5, −28.9–8.7], n = 16	0.2744
% in REM, median(IQR, Q1–Q3)	18.2 [10.3, 13.5–23.8], n = 17	14.8 [6.4, 12.6–19.0], n = 17	−1.2 [11.6, −7.4–4.2], n = 17	0.1352
Number of arousals, median(IQR, Q1–Q3)	151.0 [88.0, 99.0–187.0], n = 17	104.0 [102.0, 49.0–151.0], n = 17	−47.0 [97.0, −77.0–20.0], n = 17	0.0460
Arousal index, median(IQR, Q1–Q3)	29.6 [15.3, 19.8–35.1], n = 17	22.8 [17.4, 11.2–28.6], n = 17	−9.1 [14.4, −15.0–−0.6], n = 17	0.0638
Number of apneas, median(IQR, Q1–Q3)	37.0 [78.5, 18.5–97.0], n = 16	3.0 [19.0, 1.0–20.0], n = 17	−21.0 [80.0, −91.0–−11.0], n = 16	0.0110
Number of obstructive apneas, median(IQR, Q1–Q3)	36.0 [80.0, 16.0–96.0], n = 17	3.0 [19.0, 1.0–20.0], n = 17	−15.0 [64.0, −71.0–−7.0], n = 17	0.0040
Number of hypoapneas, median(IQR, Q1–Q3)	62.0 [57.0, 23.0–80.0], n = 17	29.0 [59.0, 12.0–71.0], n = 17	0.0 [72.0, −51.0–21.0], n = 17	0.7332
Mean oxygen saturation, median(IQR, Q1–Q3)	95.0 [2.0, 94.0–96.0], n = 18	95.0 [2.0, 94.0–96.0], n = 17	0.0 [1.0, 0.0–1.0], n = 17	0.6152
Minimum oxygen saturation, median(IQR, Q1–Q3)	82.5 [7.0, 79.0–86.0], n = 17	86.0 [9.0, 80.0–89.0], n = 17	4.0 [10.0, −2.0–8.0], n = 17	0.2682
Sleep time with oxygen saturation below 90%, median(IQR, Q1–Q3)	0.7 [3.2, 0.2–3.4], n = 16	0.4 [3.3, 0.0–3.3], n = 17	−0.2 [1.5, −1.4–0.1], n = 16	0.3757
Supine total AHI, median(IQR, Q1–Q3)	47.5 [31.1, 26.4–57.5], n = 17	12.6 [21.8, 4.6–26.4], n = 15	−24.2 [41.4, −48.0–−6.6], n = 15	0.0103
Supine REM AHI, median(IQR, Q1–Q3)	52.6 [45.8, 26.5–72.3], n = 11	18.4 [31.2, 4.0–35.2], n = 12	−32.3 [75.0, −72.3–2.7], n = 10	0.0840
Supine NREM AHI, median(IQR, Q1–Q3)	44.7 [31.9, 19.7–51.6], n = 14	26.7 [53.4, 0.0–53.4], n = 2	11.1 [0.0, 11.1, −11.1], n = 1	1.000
Off supine total AHI, median(IQR, Q1–Q3)	7.5 [5.5, 4.5–10.0], n = 17	6.7 [10.0, 4.5–14.5], n = 9	0.2 [11.8, −4.3–7.5], n = 9	0.6523
Off supine REM AHI, median(IQR, Q1–Q3)	16.1 [19.2, 7.1–26.3], n = 14	0.0 [24.7, 0.0–24.7], n = 6	−7.1 [15.8, −10.0–5.8], n = 5	0.6250
Off supine NREM AHI, median(IQR, Q1–Q3)	6.1 [6.2, 3.9–10.1], n = 14	55.5 [0.0, 55.5–55.5], n = 1	48.4 [0.0, 48.4–48.4], n = 1	1.000
Total AHI, median(IQR, Q1–Q3)	22.6 [13.9, 16.6–30.5], n = 18	4.5 [5.3, 3.0–8.3], n = 17	−17.6 [11.1, −22.3–−11.2], n = 17	0.0011
Total REM AHI, median(IQR, Q1–Q3)	28.3 [30.2, 20.7–50.9], n = 16	11.6 [25.1, 0.0–25.1], n = 11	−18.5 [31.9, −37.3–−5.4], n = 11	0.0244
Total NREM AHI, median(IQR, Q1–Q3)	19.6 [15.2, 10.9–26.1], n = 15	54.2 [0.0, 54.2–54.2], n = 1	21.4 [0.0, 21.4–21.4], n = 1	1.000
ESS, median(IQR, Q1–Q3)	7.5 [6.5, 4.5–11.0], n = 16	5.5 [5.0, 4.0–9.0]	−1.5 [4.0, −4.0–0.0], n = 16	0.0625

**Table 5:** Sleep study variables for pre- and post-treatment in NPD-OSA (n = 9 of the 27 patients with positional data).

Variable	Pre-Treatment (n = 9)	Post-Treatment (n = 9)	Difference Calculation (Post–Pre Treatment) (n = 9)	*p* Value
Total sleep time, median(IQR, Q1–Q3), n = 29	344.0 [49.0, 305.0–354.0], n = 9	357.0 [75.0, 300.0–375.0], n = 9	38.0 [67.0, 3.0–70.0]	0.5703
Sleep efficiency, median(IQR, Q1–Q3)	89.0 [6.9, 84.0–90.9], n = 9	79.3 [7.0, 75.0–82.0], n = 9	−2.8 [13.8, −10.7–3.1]	0.4961
Total awakenings, median(IQR, Q1–Q3)	17.0 [19.0, 9.0–28.0], n = 7	34.0 [24.0, 18.0–42.0], n = 7	8.0 [23.0, 7.0–30.0], n = 7	0.0156
Total time awake after sleep onset, median(IQR, Q1–Q3)	22.8 [62.3, 17.0–79.3], n = 8	65.5 [51.0, 36.0–87.0], n = 8	31.2 [38.5, 9.0–47.5], n = 8	0.0547
Sleep latency, median(IQR, Q1–Q3)	9.0 [11.5, 6.0–17.5], n = 8	11.8 [7.5, 10.0–17.5], n = 8	−1.2 [17.3, −10.5–6.8], n = 8	0.6875
REM latency, median(IQR, Q1–Q3)	145.0 [98.0, 92.0–190.0], n = 9	72.5 [153.8, 35.8–189.5], n = 8	−30.2 [196.0, −125.0–71.0], n = 8	0.7422
Total amount in supine, median(IQR, Q1–Q3)	76.0 [23.5, 58.4–81.9], n = 7	31.3 [52.0, 18.9–70.9], n = 7	−33.6 [17.8, −46.9–−29.1], n = 7	0.0313
% in REM, median(IQR, Q1–Q3)	13.7 [4.6, 12.1–16.7], n = 9	14.8 [18.3, 1.5–19.8], n = 9	−0.7 [16.5, −13.7–2.8]	1.000
Number of arousals, median(IQR, Q1–Q3)	176.0 [127.0, 114.0–241.0], n = 7	176.0 [48.0, 160.0–208.0], n = 9	0.0 [125.0, −51.0–74.0], n = 7	1.000
Arousal index, median(IQR, Q1–Q3)	32.0 [21.6, 26.2–47.7], n = 8	36.3 [5.6, 32.6–38.2], n = 9	−0.4 [18.8, −10.5–8.3], n = 8	0.9453
Number of apneas, median(IQR, Q1–Q3)	15.5 [43.5, 1.0–44.5], n = 8	0.0 [0.0, 0.0–0.0]	−15.5 [35.0, −36.0–−1.0], n = 8	0.0156
Number of obstructive apneas, median(IQR, Q1–Q3)	15.0 [29.0, 1.0–30.0], n = 9	0.0 [0.0, 0.0–0.0]	−15.0 [29.0, −30.0–−1.0]	0.0156
Number of hypoapneas, median(IQR, Q1–Q3)	94.0 [61.0, 85.0–146.0], n = 9	27.0 [75.0, 21.0–96.0], n = 9	−77.0 [60.0, −104.0–−44.0]	0.1641
Mean oxygen saturation, median(IQR, Q1–Q3)	94.0 [2.0, 92.0–94.0], n = 6	95.0 [1.0, 95.0–96.0], n = 9	1.5 [1.0, 1.0–2.0], n = 6	0.1250
Minimum oxygen saturation, median(IQR, Q1–Q3)	79.5 [16.5, 69.5–86.0], n = 8	83.0 [10.0, 79.0–89.0], n = 9	1.0 [20.0, −6.5–13.5], n = 8	0.5781
Sleep time with oxygen saturation below 90%, median(IQR, Q1–Q3)	6.0 [8.4, 0.7–9.1], n = 7	0.7 [1.0, 0.4–1.4], n = 9	−2.6 [9.2, −7.8–1.4], n = 7	0.2188
Supine total AHI, median(IQR, Q1–Q3)	34.0 [33.4, 21.2–54.6]. n = 9	10.8 [13.4, 7.6–21.0], n = 8	−22.5 [27.0, −39.3–−12.3], n = 8	0.0078
Supine REM AHI, median(IQR, Q1–Q3)	38.2 [26.0, 29.4–55.4], n = 6	4.6 [10.9, 0.0–10.9], n = 6	−29.0 [14.8, −42.4–−27.7], n = 4	0.1250
Supine NREM AHI, median(IQR, Q1–Q3)	22.9 [40.5, 14.1–54.6], n = 7	0.0 [0.0, 0.0–0.0], n = 1	−13.3 [0.0, −13.3–−13.3], n = 1	1.000
Off supine total AHI, median(IQR, Q1–Q3)	30.9 [20.5, 13.5–34.0], n = 7	1.0 [6.5, 0.0–6.5], n = 6	−11.1 [22.1, −31.2–−9.1], n = 6	0.2188
Off supine REM AHI, median(IQR, Q1–Q3)	37.1 [29.1, 15.6–44.7], n = 4	0.0 [0.0, 0.0–0.0], n = 4	−21.6 [43.1, −43.1–0.0], n = 2	1.000
Off supine NREM AHI, median(IQR, Q1–Q3)	21.6 [24.0, 10.0–34.0], n = 6	0.0 [0.0, 0.0–0.0], n = 1	−10.0 [0.0, −10.0–−10.0], n = 1	1.000
Total AHI, median(IQR, Q1–Q3)	32.0 [24.0, 16.8–40.8]	4.1 [13.6, 0.6–14.2], n = 8	−18.2 [25.4, −40.2–−14.9], n = 8	0.0078
Total REM AHI, median(IQR, Q1–Q3)	45.6 [19.0, 30.0–49.0], n = 6	0.8 [2.9, 0.0–2.9], n = 6	−40.4 [16.3, −46.3–−30.0], n = 5	0.0625
Total NREM AHI, median(IQR, Q1–Q3)	30.4 [36.8, 13.1–49.9], n = 6	0.0 [0.0, 0.0–0.0], n = 1	−12.6 [0.0, −12.6–−12.6], n = 1	1.000
ESS, median(IQR, Q1–Q3)	10.0 [6.5, 6.0–12.5], n = 8	6.0 [10.0, 2.0–12.0]	−3.0 [5.5, −6.5–−1.0], n = 8	0.0156

**Table 6:** Comparison of AHI Metrics between Positional and Non-Positional OSA Patients (n = 27 with positional data).

AHI Metric	Timepoint	NPD-OSA (n = 9)	PD-OSA (n = 18)	*p*-Value (NPD vs. PD)
**Supine AHI**	Pre	34.0 [21.2–54.6]	47.5 [26.4–57.5]	0.35
	Post	10.8 [7.6–21.0]	12.6 [4.6–26.4]	0.65
	Δ (Post–Pre)	−22.5 [−39.3 to −12.3]	−24.2 [−48.0 to −6.6]	0.97
**Off-supine AHI**	Pre	30.9 [13.5–34.0]	7.5 [4.5–10.0]	0.0021
	Post	1.0 [0.0–6.5]	6.7 [4.5–14.5]	0.17
	Δ (Post–Pre)	−11.1 [−31.2 to −9.1]	0.2 [−4.3 to 7.5]	0.0216
**Total AHI**	Pre	32.0 [16.8–40.8]	22.6 [16.6–30.5]	0.46
	Post	4.1 [0.6–14.2]	4.5 [3.0–8.3]	0.70
	Δ (Post–Pre)	−18.2 [−40.2 to −14.9]	−17.6 [−22.3 to −11.2]	0.35
**Total REM AHI**	Pre	45.6 [30.0–49.0]	28.3 [20.7–50.9]	0.25
	Post	0.8 [0.0–2.9]	11.6 [0.0–25.1]	0.21
	Δ (Post–Pre)	−40.4 [−46.3 to −30.0]	−18.5 [−37.3 to −5.4]	0.0699

## Data Availability

The data presented in this study are available on request from the corresponding author due to privacy restrictions.

## Abbreviations

The following abbreviations are used in this manuscript:

OSA	Obstructive Sleep Apnea
HNS	Hypoglossal Nerve Stimulation
PD-OSA	Position-Dependent Obstructive Sleep Apnea
NPD-OSA	Non-Position Dependent Obstructive Sleep Apnea
REM	Rapid Eye Movements
NREM	Non-Rapid Eye Movements
